# Knowledge, Attitude, and Practice Regarding Diabetes Mellitus Among Type 2 Diabetic Patients Attending Primary Health Care Centers in the Jazan Region of Saudi Arabia

**DOI:** 10.7759/cureus.28704

**Published:** 2022-09-02

**Authors:** Mohammad A Mahzari, Omar H Oraibi, Ayman M Shami, Mohammed O Shami, Tayeb Y Thobab, Abdulaziz A Awlaqi, Roaa A Abu Allah, Fahad Y Azyabi, Faisal Otaif, Khalid Majrashi, Ahmed K Alwan, Mohammed H Hazazi

**Affiliations:** 1 Public Health, Chest Disease Hospital, Jazan, SAU; 2 Internal Medicine/Endocrinology/Diabetes and Metabolism, Jazan University, Jazan, SAU; 3 College of Medicine, Jazan University, Jazan, SAU; 4 Family Medicine, Abu Arish Primary Health Care Center, Jazan, SAU

**Keywords:** diabetes mellitus, knowledge, and practice, attitude, dm: diabetes mellitus

## Abstract

Background

Diabetes mellitus is a disease whose dramatic increase in prevalence worldwide poses a global health crisis. Saudi Arabia has the seventh highest rate of diabetes in the world and the second highest rate in the Middle East. The incidence of diabetes has risen due to insufficient knowledge, attitude, and practice surrounding the disease. Saudis’ quality of life has decreased due to the recent rise in diabetes-related mortality and illnesses. Hence, leading healthy lives requires patients to have positive attitudes and self-awareness, which will eventually contribute to reducing diabetes mellites-related complications.

Methods

A cross-sectional study among patients with type 2 diabetes mellites (T2DM) in the Jazan region of Saudi Arabia was conducted to assess Saudi adults’ knowledge, attitude, and practice (KAP) regarding the disease. The patients participated in the study by filling in a questionnaire. Data analysis was carried out using R software, version 4.0.5 (R Studio: Integrated Development for R, Boston, MA) and the analysis included general sample demographics. KAP scores were the dependent variables. Following the sample description, regression analysis was performed to examine the adjusted relationships between KAP factors and independent variables. The impact of all independent variables on KAP variables was examined using multiple linear regression analysis. A p-value of less than 0.05 was considered significant, and the beta coefficient was applied to estimate the associations between the independent variables and the KAP variables.

Results

A total of 424 participants were involved in this study, with 194 male participants and 230 female participants. The mean duration of having T2DM was 7.63 ± 7.19 years. More than half of the study participants had a degree (58.49%), and nearly half were employed (42.45%). Married participants had higher knowledge and attitude scores than single and divorced or widowed participants (9.19 ± 3.38, 80.14 ± 14.72, respectively). In comparison, divorced or widowed participants had higher practice scores than single and married participants (16.35 ± 7.42). Participants with higher education attainment had higher knowledge and attitude scores than those with no degree (9.54 ± 3.39, 80.58 ± 17.57, respectively), whereas participants with no degree demonstrated higher practice scores (16.85 ± 8.3) than those with degrees. In examining the study participants’ KAP scores, we found knowledge to be insufficient in 51.2% and sufficient in 48.8% of the participants. Of the participants, 7.8% were found to have negative attitude levels and 92.2% were found to have positive attitude levels. Finally, study participants’ practice levels were found to be negative in 24.8% and positive in 75.2% of participants.

Conclusions

This study revealed that patients have gaps in their knowledge, attitude, and practice in regard to T2DM. In this study, associations and correlations were established between KAP scores and the sociodemographic characteristics of the patients. The findings of this study could be helpful to policymakers, decision-makers, health care professionals, and patient advocacy groups in developing interventions to improve the health outcomes in T2DM patients.

## Introduction

Diabetes mellitus (DM) is a chronic metabolic condition characterized by unusually high blood glucose levels. Blood sugar levels stay high in those with diabetes; the most prevalent forms are type 1 DM (T1DM; 5%) and type 2 DM (T2DM; 95%), which are brought on by problems with the pancreas’ production of insulin or by the body’s inability to effectively use the insulin it produces [1,2]. Saudi Arabia has the seventh highest rate of DM in the world and the second highest rate in the Middle East [3]. According to a study, there are an estimated 3 million prediabetics and 7 million diabetics in Saudi Arabia [4].

Saudis’ quality of life has decreased as a result of the recent rise in diabetes-related mortality, illnesses, and general frailty among T2DM patients (WHO, 2010). Leading a healthy life requires patients to have positive attitudes and self-awareness. Patients who are aware of the difficulties of diabetes seek out the right care and treatment and maintain their health [5].

Both plasma insulin and C-peptide concentrations are often elevated in people with T2DM, demonstrating underlying insulin resistance [2]. Despite insulin resistance, chronic hyperglycemia can nevertheless result in a temporary insulin deficit (also known as glucose toxicity) with a low initial plasma insulin concentration. Therefore, if there is acute metabolic impairment at the presentation, insulin, and C-peptide levels may be deceptively low [6]. A hemoglobin (Hb)A1c level of 6.5% or higher; or a fasting plasma glucose level of 126 mg/dL (7.0 mmol/L) or higher (fasting is defined as no calorie intake for at least 8 hours) a 2-hour plasma glucose level of 200 mg/dL (11.1 mmol/L) or higher during a 75 g oral glucose tolerance test or random plasma glucose of 200 mg, these levels are indicators of diabetes [7]. Symptoms of diabetes include heightened thirst, weakness, exhaustion, distorted vision, tingling or numbness in the hands or feet, slow-healing cuts or sores, unanticipated weight loss, frequent urination, frequent infections without a known cause, and mouth aches [8].

Management of T2DM includes dietary modifications such as monotherapy or dietary modifications with metformin [6,8]. Treatment should be escalated by adding a second drug if the HbA1c target has not been reached within around three months of beginning the initial medication. If the HbA1c target is not met, triple therapy should be explored, and glycemic control should be evaluated once more in about three months. To establish glycemic control, combination injectable therapy with basal insulin may be used if the HbA1c target is still not met. Dual-combination therapy as the first line of treatment is an option for patients with high baseline HbA1c values. Although the American Diabetes Association (ADA) advises considering starting dual therapy if the entry HbA1c level is 9%, the American Association of Clinical Endocrinologists and American College of Endocrinology recommends initial dual therapy (i.e., metformin plus another agent in addition to lifestyle management) for patients with an entry HbA1c level of 7.5% [9,10].

The rate of diabetes has risen due to insufficient knowledge, attitude, and practice [3]. It is crucial that everyone in Saudi Arabia is aware of the factors that lead to diabetes, such as cigarette use, unhealthy diet, lack of exercise, being overweight, and obesity. Studies conducted in the Jazan region in Saudi Arabia have revealed that the physical, psychological, social, and environmental components of quality of life were all negatively affected by the stigma of being overweight [11,12]. Through education and better information, patients themselves can lessen and regulate these negative effects [13].

Patients with diabetes should be well-versed in explicit knowledge, attitude, and practice to prevent and control complications related to their condition. Knowledge, attitude, and practice are crucial to preventing and limiting the effects of diabetes [14].

We can more successfully improve diabetes awareness and education by being aware of the degree of knowledge, attitude, and practice among those with the disease. As a result, patient education, attitude change, and dietary practice can all be improved by proper education and awareness programs developed in accordance with society’s needs [15]. This study aims to evaluate the prevalence of T2DM as well as patient knowledge, attitudes, and practices in the Jazan region of Saudi Arabia. Furthermore, the relationship between social determinants and T2DM is evaluated.

## Materials and methods

Study design and participants

A cross-sectional, descriptive study was conducted between February 2022 and July 2022 in the Jazan region, which lies in the southwest corner of Saudi Arabia and has a population of 1,365,110, according to the 2010 census. A sample of 384 participants (minimum sample size) was calculated using Epi Info, version 7 (Centers for Disease Control and Prevention, Atlanta, USA), with a confidence interval of 95%, a 5% allowed error, an expected prevalence rate of the knowledge to be 50%, and population size of 1,365,110. A total of 424 participants were included in our study. The inclusion criteria were Saudi males and females aged 18 years and above attending the selected primary health care centers during the study. We invited the patients to participate in the study by filling out the questionnaire themselves after explaining how to fill it out. The researchers were in the room with the participants as they filled in the questionnaire answered any questions and provided clarifications. The researchers and trained assistant nurses collected the questionnaires. The principal researcher supervised the overall data collection activities.

Measures

Sociodemographic Variables

The first section of the survey included questions about the participant’s demographics, such as their gender, age, occupation, marital status, educational level, income, family history of diabetes, duration of diabetes, and medications.

Knowledge, Attitude, and Practice (KAP) Questionnaire

The second section consisted of the knowledge, attitude, and practice (KAP) questionnaire obtained with permission from the Diabetes Research and Training Center of Michigan. This questionnaire had a series of valid and reliable questions that could be used in KAP assessments. The instrument was modified and translated into Arabic by an expert. The KAP questionnaire comprised 61 questions (knowledge: 20; attitude: 22; and practice: 19). KAP scores were computed such that correct answers were assigned a score of 1, whereas wrong or “not sure” answers were assigned a score of 0. A total score was computed for every participant, and percentages of the total KAP scores were computed. Those with 50% or more were considered to have sufficient knowledge, positive attitude, and adequate practice, whereas those with less than 50% were considered to have “insufficient knowledge or negative attitude, and inadequate practice.

Statistical analysis

Data analysis was carried out using R software, version 4.0.5 (R Studio: Integrated Development for R, Boston, MA). The analysis started with general sample characteristics, including descriptive statistics for the data variables. Sex, age, marital status, education, employment, income, years with T2DM, and relatives with T2DM were the independent variables. KAP scores were the dependent variables. The frequency distributions for categorical variables and summary statistics for continuous variables were then provided. Following the sample description, regression analysis was performed to examine the adjusted relationships between KAP factors and independent variables. The impact of all independent variables on KAP variables was examined using multiple linear regression analysis. A p-value of less than 0.05 was considered significant, and the beta coefficient was applied to measure the associations between the independent variables and the KAP variables. 

Ethical considerations

The study was ethically approved by the Jazan Health Ethics Committee (approval number 2218, dated 15/2/2022). All procedures performed in our study involving human participants followed the ethical standards of the institutional and/or national research committee and with the 1964 Helsinki Declaration and its later amendments or comparable ethical standards.

## Results

Table 1 shows a detailed description of the baseline characteristics of study participants. A total of 424 participants were involved in this study. Among the 424 participants, 194 (45.75%) were male, and 230 (54.25%) were female. The mean age was 40.72 years ± 15.97 years, and the mean duration of having T2DM was 7.63 years ± 7.19 years. Most of the study participants (61.55%) were married, 32.31% were single, and 6.13% were divorced or widowed. More than half of the study participants were educated (58.49%), and nearly half were employed (42.45%). Most of the participants (59.2%) had relatives with T2DM.

**Table 1:** Baseline characteristics of the sample (n = 424) SD = Standard deviation; T2DM = Type 2 diabetes mellitus

Characteristics	Mean (SD)
Age (years)	40.72 ± 15.97
Duration of T2DM	7.63 ± 7.19
Characteristics	Frequency (%)
Sex	
Male	194 (45.75%)
Female	230 (54.25%)
Marital Status	
Married	261 (61.55%)
Single	137 (32.31%)
Divorced/Widowed	26 (6.13%)
Education	
Has Degree	248 (58.49%)
No Degree	176 (41.51%)
Employment	
Employed	180 (42.45%)
Unemployed	244 (57.55%)
Income (Saudi Riyal)	
0–4,999	133 (31.37%)
5,000–9,999	116 (27.36%)
10,000–14,999	86 (20.28%)
15,000–19,999	66 (15.57%)
≥20,000	23 (5.53%)
Relatives with T2DM	
Yes	251 (59.2%)
No	173 (40.8%)

Table 2 shows KAP scores among study participants. Participants’ knowledge about T2DM was represented by their knowledge score. For the whole sample, the respondents’ mean (±SD) KAP scores were 8.89 ± 3.6, 79.03 ± 16.44, and 15.81 ± 8.2, respectively. The mean KAP scores were higher among males than females, the mean knowledge score was 9.44 ± 3.81, the mean attitude score was 79.07 ± 13.71, and the mean practice score was 15.89 ± 8.4. Regarding marital status, married participants had higher knowledge and attitude scores than single and divorced or widowed participants (9.19 ± 3.38, 80.14 ± 14.72, respectively). In comparison, divorced or widowed participants demonstrated higher practice scores than single and married participants (16.35 ± 7.42). Participants with higher education attainment had higher knowledge and attitude scores than those with no degree (9.54 ± 3.39, 80.58 ± 17.57, respectively), whereas participants with no degree had higher practice scores (16.85 ± 8.3). Employed participants had higher knowledge and attitude scores than unemployed participants (9.46 ± 3.5, 80.65 ± 16.51, respectively), whereas unemployed participants had higher practice scores (16.25 ± 8.42). Regarding income, participants with incomes ≥ 20,000 SR (Saudi Riyal) had high knowledge scores (10.22 ± 2.89), those with incomes 10,000-14,999 SR had high attitude scores (81.45 ± 16.27), and those with incomes 5,000-9,999 SR had high practice scores (17.47 ± 8.4). Participants with relatives with T2DM demonstrated higher KAP scores (9.24 ± 3.21, 79.56 ± 14.89, 16.46 ± 8.26, respectively).

**Table 2:** Participants’ KAP scores KAP: Knowledge, attitude, and practice, T2DM: Type 2 diabetes mellitus

Variables		Knowledge (mean ± SD)	Attitude (mean ± SD)	Practice (mean ± SD)
Whole Sample	N=424	8.89 ± 3.6	79.03 ± 16.44	15.81 ± 8.2
Sex	Male	9.44 ± 3.81	79.07 ± 13.71	15.89 ± 8.4
	Female	8.43 ± 3.35	79 ± 18.47	15.75 ± 8.04
Marital Status	Married	9.19 ± 3.38	80.14 ± 14.72	16.22 ± 8.39
	Single	8.66 ± 3.96	78.16 ± 19.31	14.94 ± 7.94
	Divorced/Widowed	7.12 ± 3.28	72.46 ± 15.2	16.35 ± 7.42
Education	Has Degree	9.54 ± 3.39	80.58 ± 17.57	15.08 ± 8.06
	No Degree	7.98 ± 3.7	76.85 ± 14.48	16.85 ± 8.3
Employment	Employed	9.46 ± 3.5	80.65 ± 16.51	15.22 ± 7.87
	Unemployed	8.47 ± 3.62	77.84 ± 16.32	16.25 ± 8.42
Income	0–4,999	8.63 ± 3.69	76.86 ± 13.52	16.62 ± 8.14
	5,000–9,999	8.8 ± 3.48	80.1 ± 16.28	17.47 ± 8.4
	10,000–14,999	8.95 ± 3.3	81.45 ± 16.27	15.1 ± 8.08
	15,000–19,999	9.03 ± 4.18	79.24 ± 21.2	12.8 ± 7.97
	≥20,000	10.22 ± 2.89	76.48 ± 17.4	14.09 ± 5.88
Relatives with T2DM	Yes	9.24 ± 3.21	79.56 ± 14.89	16.46 ± 8.26
	No	8.39 ± 4.07	78.26 ± 18.49	14.88 ± 8.04

**Figure 1:**
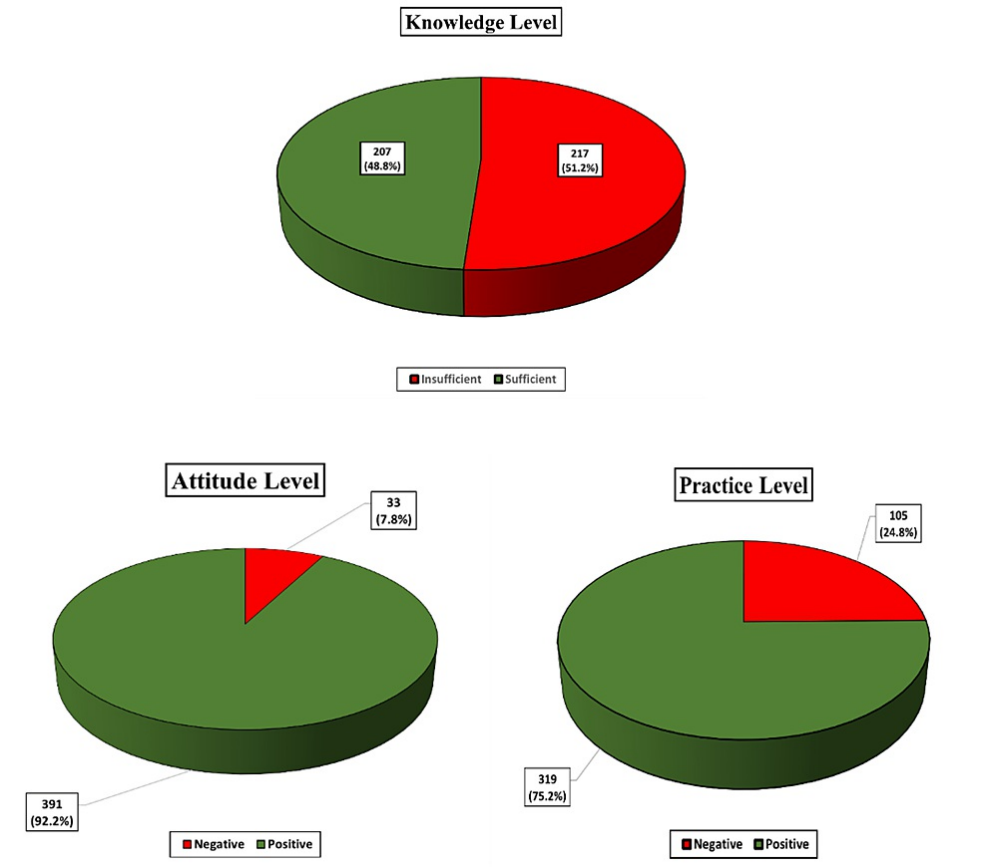
Participants’ KAP levels KAP: Knowledge, attitude, and practice

Figure 1 shows the breakdown of study participants’ KAP levels. Among all participants, the levels of knowledge were found to be insufficient in 51.2% and sufficient in 48.8% of the study participants. Of the participants, 92.2% had positive attitudes, whereas 7.8% had negative attitudes. Finally, practice levels were negative in 24.8% and positive in 75.2% of the study participants.

Multiple linear regression analysis was performed to examine the relationship between the sample’s KAP variables and sociodemographic characteristics. Longer diabetes duration was positively and significantly associated with higher knowledge and practice scores (beta coefficient {B} = 0.10, p < 0.001, B = 0.26, p < 0.001, respectively). Similarly, participants who had first-degree relatives with T2DM had a higher knowledge score compared to individuals with no relatives with T2DM (B = 0.78, p = 0.028). Conversely, participants with no academic degree had a lower knowledge score compared to individuals with a degree (e.g., bachelor’s/master’s degree or PhD; B = -1.66, p < 0.001). Additionally, older individuals had significantly lower practice scores (B = -0.08, p = 0.042). Furthermore, widowed and divorced participants had substantially lower attitude scores than married and single participants (B = -8.17, p = 0.033). Finally, having an income between 15,000 and 19,999 SR was significantly associated with low practice scores (B = -3.85, p = 0.004). The rest of the associations are summarized in Table 3.

**Table 3:** Association between KAP scores and sociodemographic characteristics Linear regression analysis of the associations between KAP scores and sociodemographic characteristics KAP: Knowledge, attitude, and practice, B: Beta coefficient, *: p-value < 0.05

Variables	Levels	Knowledge (B + p-value)	Attitude (B + p-value)	Practice (B + p-value)
Sex (Reference: Female)	Male	0.55 (0.133)	-1.20 (0.491)	0.53 (0.530)
Age		-0.003 (0.818)	0.10 (0.174)	-0.08 (0.042) *
Marital Status (Reference: Married)	Single	-0.04 (0.933)	0.07 (0.979)	-1.82 (0.136)
	Divorced/widowed	-1.30 (0.106)	-8.17 (0.033) *	0.47 (0.803)
Education (Reference: Has Degree)	No Degree	-1.66 (<0.001) *	-3.04 (0.135)	0.33 (0.736)
Employment (Reference: Employed)	Unemployed	-0.41 (0.343)	-1.51 (0.460)	0.65 (0.518)
Income (Reference: 0–4,999)	5000-9999	-0.31 (0.502)	1.93 (0.382)	0.45 (0.672)
	10000-14999	-0.59 (0.270)	3.03 (0.234)	-1.60 (0.197)
	15000-19999	-0.87 (0.131)	0.09 (0.974)	-3.85 (0.004) *
	>20000	0.67 (0.413)	-2.43 (0.531)	-2.05 (0.279)
Years with T2DM		0.10 (<0.001) *	-0.21 (0.093)	0.26 (<0.001) *
Relatives with T2DM (Reference: No)	Yes	0.78 (0.028) *	2.24 (0.181)	1.2 (0.222)

## Discussion

DM is a major public health issue in Saudi Arabia, resulting in high morbidity and mortality rates. According to the World Health Organization (WHO), Saudi Arabia has the second-highest diabetes rate in the Middle East and is ranked seventh in the world [3]. According to a local cross-sectional study by Alqurashi et al. [16], 30% of the Saudi population is diabetic; among those with diabetes, 34.1% are men. It is widely accepted that patient education and involvement can help patients better manage their disease [17,18]. The ADA has highlighted the need for clinical care, self-care practices, and patient education to manage and prevent chronic consequences of such a community health problem. This cross-sectional study assessed the KAP scores among patients with T2DM in Jazan, Saudi Arabia. More than half of the participants in our study showed insufficient knowledge about T2DM. Attitude and practice levels were also determined to be positive in most of the participants.

Our study participants demonstrated insufficient overall knowledge, which contradicts numerous other studies [19-22] but is similar to Al-Aboudi et al.’s findings [23]. This study’s participants showed a positive attitude and practice regarding T2DM, similar to Abougalambou et al. [19], who found that 87.7% of their participants had a good attitude. However, our results showed more than half of the participants showed poor practice. As expected, participants with relatives with T2DM had higher KAP scores, which can be explained by the fact that having relatives with a similar condition will enhance one’s knowledge of that condition because they will share their knowledge and experience.

In the present study, being male, having high education attainment, and being employed were correlated with higher knowledge scores. Other studies have obtained different results regarding knowledge of T2DM. The average knowledge score for DM was good among the participants in Mansy et al.'s [24] and Zibran et al.’s [25] studies. Conversely, in India, Srinivasan et al. [26] showed that 58% of their participants lacked adequate knowledge of T2DM. Changes in the participants’ sociodemographic characteristics, educational levels, and the amount of diabetic knowledge accessible at the time might explain the discrepancies in the participants’ level of knowledge of DM between studies. We recommend using social media websites to enhance the knowledge of T2DM among diabetic patients. A diabetes educational program should be implemented to improve patients’ attitudes and practices toward diabetes.

This study revealed that patients have gaps in their knowledge, attitude, and practice in regard to T2DM. In this study, associations and correlations were established between the patients’ KAP scores and their sociodemographic characteristics. The findings of this study may be helpful to policymakers, decision-makers, health care professionals, and patient advocacy groups in developing interventions to improve the health outcomes of T2DM patients.

## Conclusions

The study results showed that patients have gaps in their knowledge, attitude, and practice regarding T2DM. Among all participants, the levels of knowledge were found to be insufficient in 51.2% and sufficient in 48.8% of the study participants. Out of the total, 92.2% of the participants had positive attitudes, whereas 7.8% had negative attitudes. Finally, practice levels were negative in 24.8% and positive in 75.2% of the study participants. Furthermore, longer diabetes duration and having first-degree relatives with T2DM were positively and significantly associated with higher knowledge scores.

We recommend using social media platforms to enhance the knowledge of T2DM among diabetic patients. To improve patients’ attitudes and practices toward diabetes, a specific diabetes education program aiming to raise knowledge, attitude, and practices toward type 2 diabetes mellitus targeting people with diabetes and their close family members and caregivers should be implemented.
